# When problems just bounce back: about the relation between resilience and academic success in German tertiary education

**DOI:** 10.1007/s43545-021-00060-6

**Published:** 2021-02-15

**Authors:** Felix Bittmann

**Affiliations:** Leibniz Institute for Educational Trajectories, Wilhelmsplatz 3, 96047 Bamberg, Germany

**Keywords:** Psychological resilience, Tertiary education, Germany, Panel analyses

## Abstract

While in the past the concept of resilience was used to explain psychological well-being in extreme situations like enduring poverty, abuse, or war, it has now found broad application in numerous fields of research. It can also be applied to examine how everyday challenges and problems are dealt with, for example in the educational and university context. This raises the question of whether resilience and academic success are correlated. Using German longitudinal data including university and university of applied sciences students in their first four years (2010–2015) we investigate how resilience and various measurements of success (satisfaction, intention to drop out, grades) are correlated using multilevel growth-curve models. We demonstrate that resilient individuals have consistently more positive academic trajectories, have lower dropout intentions, report better grades and are more satisfied with their lives. The effects are exceptionally stable over time, statistically highly significant and of considerable magnitude. This demonstrates that resilience is associated with better outcomes in university students under control of a large number of potential confounding factors and influences.

## Introduction

While in the past the concept of resilience was used primarily to explain psychological resilience in disasters and in emergency situations, for example in war or extreme poverty (Luthar 1999), it is now also utilized to explain how to deal with everyday psycho-social conflicts and problems. The term is particularly fruitful when psychologically challenging events or phases of life are of interest, such as late adolescence and the transition to tertiary education. This is where various risk factors come together: On the one hand, this phase of life is characterized by a number of serious changes, such as leaving the parental home, running one's own household, decay and the formation of new social networks and growing personal responsibility (Pidgeon et al. 2014). On the other hand, integration into the tertiary education system is taking place, which represents a considerable additional burden due to the demands of universities and other institutions. Moreover, the success of this educational phase determines the course of the entire professional career and thus ultimately a large part of the further course of life. Setbacks and problems are practically unavoidable, so that dealing constructively with these challenges is ultimately more important than simply avoiding them. This raises the question of what role resilience plays in the university context and whether students benefit from resilience in terms of academic success. Is it demonstrable that students with more pronounced resilience are better able to deal with negative events and are less affected by them? Do such students report more positive and more successful trajectories? On the one hand, clarification of these questions seems relevant to demonstrate that resilience is a significant concept with practical implications not only in extreme situations but also in everyday life. On the other hand, in this case it would also be useful to further investigate when and how resilience is developed and whether it can be learned and trained in order to have a protective factor against negative life events.

In summary, the following research question is at focus: Do students in tertiary education with and without resilience status differ in their trajectories, for example in terms of the tendency to drop out, the grades achieved in their studies or their general life satisfaction?

## Theoretical considerations

Although there is no universal definition of resilience, it is generally regarded as the ability of an individual to cope with challenging phases of life and to adapt successfully to the environment (Wagnild and Young 1993). Other, more sophisticated definitions sometimes emphasize the process character of resilience (Masten et al. 1990). Here, resilience is not seen as a constant characteristic of the individual, but rather as a time-variable component that allows interaction between the individual and the environment. In general, a meta-study reviewing several decades of resilience literature identifies two central core themes that are common to most definitions: *adversity* and *adaption* (Fletcher and Sarkar 2013). By this, the authors mean the emergence of negative events in the life course of an individual that requires adaption. These approaches, which are undoubtedly well-founded and relevant, are often hardly applicable for empirical studies, since it is difficult to adequately map or measure these interactions even in longitudinal studies, that is, conceptualizing and measuring both the adversity and the reaction or adaption of the individual. Owing to these practical limitations, resilience is then often understood as a relatively stable personality disposition whose origins and development presumably take place in (early) childhood (Werner 2008, pp. 22–25) and are largely completed in late adolescence (Ong et al. 2009). This approach follows previous empirical research studies.^1^ A recent overview of a large number of studies and resilience programs further extends the classical definition described here and identifies other strands, which might be especially beneficial for researchers working on the development and implementation of resilience-training (IJntema et al. 2019).

How can the effect of resilience in the course of the study be explained? It should be noted that the term can only be applied meaningfully if the occurrence of stressful and psychologically challenging events is given. The term resilience is therefore not applicable if no negative or challenging experiences occur. However, it seems obvious that most college trajectories are sometimes stress-inducing and this also over longer periods of time. First of all, various transition processes at the end of late adolescence should be mentioned, such as leaving the parental home (Kenny 1987, p. 23), building up one's own lifestyle, independence and integration into an often new social environment, which can be perceived as a challenge (Kerr et al. 2004). In addition, there are the ongoing burdens of studying, which continue to exist even after successful initial adaptation to the new life situation: Mastering everyday student life, independent living, learning and examinations. Defeats and conflicts are practically unavoidable and it is not strict prevention, but rather a positive and constructive approach to these problems that determines further success. According to the preceding definition, this means that resilient persons adapt to these challenges and maintain their own well-being. The assumed advantage of resilience is therefore not primarily to be able to avoid negative events, but rather to understand them as meaningful and manageable challenges and not to be distracted from the original goals (Hartley 2011). For a more detailed explanation, the variables that are later included in order to operationalize the success of the studies should be listed: the tendency to consider to discontinue the studies, the average grades achieved in the studies and the general life satisfaction. Thus, for example, it can be assumed that, in view of the challenging events, resilient individuals nevertheless maintain a high level of satisfaction because they perceive the stressors as manageable. For the same reasons, the tendency to want to drop out of the study should be lower, since a break-off only seems inevitable if problems or challenges are perceived as overwhelming and not manageable. Finally, the average grades should also be better, since nonresilient persons are more strongly affected by negative events and, for example, concentration or stamina are negatively influenced (Talib and Zia-ur-Rehman 2012).Based on these assumptions, the following hypothesis can be formulated: Persons with a high degree of resilience have, on average, more successful courses of study compared to persons with a lower degree of resilience, i.e., they have a lower tendency to drop out, better grades and higher satisfaction (Hypothesis 1).

Overall, the hypothesis is supported by previous research results: It has been shown that resilience and well-being correlate positively (Abolghasemi and Varaniyab 2010). Furthermore, there is a positive relationship between resilience level and problem-solving skills (Coşkun et al. 2014) and high resilience positively influences the reading performance of students (Zuill 2016). Hartley (2011) shows that resilience counteracts dropout, other studies confirm the relationship between resilience and academic success (Allan et al. 2014; Esquivel et al. 2011). Another study demonstrates that university students with high levels of resilience have a more stable mental health than those with low values, which relates to several outcomes under investigation and shows the advantage for students with high resilience (Kapikiran and Acun-Kapikiran 2016). Why these positive outcomes are present is explained in more detail by a study that positive personality traits like openness, emotional stability and conscientiousness are all positively related to resilience and resilience is able to explain a positive study progress (Backmann et al. 2019). Especially in the short-term, resilience is a strong predictor of success and mental health of students (Wu et al. 2020). Since these results are from China, it can be assumed that resilience is rather independent of culture and setting and thus a general and stable predictor. Two other studies that look at the role of resilience and mediation factors come to similar conclusions as resilience is related to healthy coping strategies in the presence of challenging situations (de la Fuente et al. 2017; McLafferty et al. 2012). These findings are support by another study which investigates the association of resilience and dropout rates and finds that the chances for dropping out of the study are significantly lower in the group with high levels of resilience (Van Hoek et al. 2019). One more finding supports the overall correlation between resilience and higher levels of optimism that are related to success in university students (Gómez- Molinero et al. 2018). Thus, this overview of the literature suggests that higher levels of resilience are associated with positive outcomes like a higher success rate and lower levels of dropping out, which all support the hypothesis stated above.

Summarized, the following analyses go beyond most of the studies mentioned. First, longitudinal data are available, which means that complete academic courses are recorded and the various outcomes can be traced over time. This is a clear advantage over cross-sectional studies that only include one point of measurement, as these can only provide information on the stability of effects to a limited extent. Furthermore, there is a clear temporal order (resilience is measured before the outcomes), so that the problem of a reverse causality does not exist, in contrast to cross-sectional studies. In contrast to some previous studies, with the data at hand one can actually cover almost the entire time of a student in the tertiary system, so long-term developments become visible. Finally, in comparison to some longitudinal studies on resilience, the available data also contain comprehensive contextual information, so that relevant control variables can be included. Only very few studies have the resources to include both a large set of items and survey students repeatedly, which should enable some new insights. The present study is therefore based on comparatively comprehensive and high-quality data which are unique, at least for Germany.

## Method

### Data and sample

We use data from Germany to estimate the effects of resilience on outcomes. Data from the *National Educational Panel Stud*y (NEPS) (starting cohort 5, version 12-0-0), a large-scale assessment of educational trajectories in Germany, are suitable for analyzing the research question posed (Blossfeld et al. 2011).^2^ The sample comprises university students from all over Germany who are regularly surveyed from the beginning of their studies on their study programs, developments and grades, but also on a variety of other areas of life. The NEPS is a suitable basis as detailed information on social background and childhood is available in addition to the prospectively collected data on the trajectories, which is crucial to partial out biasing effects or self-selection. The original sample consists of 17,910 students who began their first degree at a university in Germany in the winter term 2010/2011, regardless of nationality, subject or degree. The sample is clustered by universities and subjects (104 public universities, 108 public universities of applied sciences and 49 private universities) (FDZ-LIfBi 2018). The surveys take place regularly (once a year). Students with a degree in teaching as well as students in private educational institutions are overrepresented by the design in order to provide more accurate information for these relatively small populations. Of the 31,082 persons contacted, 17,910 were finally interviewed after checking the target sample affiliation, which corresponds to a participation rate of approximately 58% (wave 1). The realization rate according to AAPOR (2006) is 85.2% (Steinwede and Aust 2012, p. 38). Overall, starting cohort 5 claims to be representative of persons studying in Germany for the first time in 2010/11. As expected, this figure will decline over time as people leave the panel temporarily or permanently (attrition). The data collection is carried out by Infas (Bonn) and the German Centre for Higher Education and Science Research (DZHW, Hanover). For the following analyses, the data are additionally restricted. In all analyses, only students who were not older than 35 years at the beginning of the data collection in wave 1 will be included in order to obtain a “typical” student sample. For example, the processes described in theory cannot be assumed in the same way for students who are retired and are studying for personal development after completing their professional careers. Moreover, in the longitudinal analyses, only data from the first four waves from 2011 to 2014 are used, since beyond that many variables are no longer available and the sample size would be considerably reduced.

### Operationalization and variables

The operationalization of the variable resilience is of central importance for all subsequent analyses. A review paper that identifies and assesses 19 different scales in the literature, but does not recommend a particular operationalization, shows how various resilience can be measured (Windle et al. 2011). A solution that is compatible with the existing data is operationalization using the Big Five Inventory. Here, resilience is generated as a binary variable from three metrically scaled inventory items (Ercan 2017). Accordingly, persons are considered resilient if they have a below-average value for neuroticism and above-average values for conscientiousness and extraversion as one study reports (Campbell-Sills et al. 2006, p. 591). The other components of the big Five inventory are not predictive of the resilience status in a multivariate model and are thus not included in the operationalization. The robustness of this form and similar forms (including all five components of the Big Five inventory) has been empirically proven (Waaktaar and Torgersen 2010). The validity of such a procedure is confirmed by other studies that investigate the connection between Big Five and study success. Thus, a meta-study involving 58 individual studies comes to the conclusion that neuroticism and satisfaction with the course of the study correlate negatively and conscientiousness and performance correlate positively (Trapmann et al. 2007). It can therefore be assumed that postulated correlations are stable overall. The resilience status can be calculated for the year 2012, since the inventory items mentioned are measured at that time. This relatively early measurement, even if not directly at the beginning of the study, is helpful, as it provides the temporal order for a better understanding in which causality flows. The respective inventory items are measured on a scale with nine levels between 1.0 and 5.0 in steps of 0.5 points. Descriptively, about 17% of all students are classified as resilient in 2012.

The variable of social origin is particularly important in order to be able to exclude spurious correlations as far as possible. There are various operationalizations for this, but parental status as a continuous variable seems more suitable than, for example, educational level, which was only measured categorically. The average parental ISEI (*International Socio-Economic Index of Occupational Status*), which is based on the respective occupation, is used. This scale has the advantage that various aspects such as income, educational level and prestige are taken into account, which allows a differentiated measurement of origin (Ganzeboom et al. 1992). If information is available for father and mother (which is the case for 84.1% of all respondents), the highest value is taken. Otherwise, the only available value was used.

After the central explanatory variables, the dependent variables will be explained. For this purpose, three time-variable outcomes were selected for which information is available in the four waves between 2011 and 2014. The first includes the tendency to drop out of the current study (Trautwein et al. 2007). For this purpose, a new quasimetric variable was generated from five different items with four steps each (Likert scaling) (Cronbach alpha > 0.79, depending on the wave). The original items ask, for example, whether the respondent has already seriously considered discontinuing the study. The relevance of the variable is that it estimates the probability that a study will be successfully completed. Students with a high dropout tendency are likely to give in to this sooner or later, which is of considerable importance for the further course of life. The second dependent variable is the self-reported average study grade in the current semester, measured on a scale of 1.0 to 4.0, with higher values representing better grades. It should be noted that this variable, like all others, was collected in the survey and cannot be verified objectively. This variable is used in an unstandardized way, but we control for the field of study. The relevance of the study grade lies in the measurement of general performance. Better grades are by definition an expression of higher academic performance. The last dependent variable is general life satisfaction, which is generated from a total of six individual items (Westermann et al. 1996). Each item is measured on an eleven-point scale between 0 and 10; higher scores represent higher satisfaction (Cronbach alpha > 0.84, depending on the wave). This variable appears relevant to reflect the general quality of life, which is of central interest to the individual. These items account for various sub-dimensions like “How satisfied are you with… your life/your health/your standard of living/your family life?” to give an impression on various forms of overall satisfaction, which can be integrated into a single combined score.

In order to select all control variables, it is useful to integrate the following analyses into a causal theoretical framework. This means that the quality of empirical research can be improved if the intention to explain causality is explicitly stated (Hernán 2018). (Pearl 2009).Based on the framework as proposed by Pearl, to rule out spurious correlations it is necessary to include all variables as controls that influence both the treatment as well as the outcome simultaneously. It is important to note that it is not the choice of statistical method for identifying causal effects that is decisive, but the selection of the relevant control variables. All those that influence the independent and dependent variables simultaneously must be selected. The following control variables are used for this purpose: Gender, age, place of birth (West Germany, East Germany, abroad), migration status, whether the study was taken up at a university or at a university of applied sciences, field of study (pre-grouped into six categories by the NEPS due to data protection regulations), highest parental ISEI, highest parental educational level, number of siblings, years spent in kindergarten, death of father, death of mother, age of both parents in 2011 and student loan eligibility. The logic of this selection is explained using the example of the parental ISEI: A higher social status goes hand in hand with greater resilience (Schoon 2006). At the same time, social status has an impact on the study situation, since parents with a high social status can provide more financial resources and thus have a positive influence on the housing and living situation during the study period as well as on life satisfaction. If the social status were not included, a spurious correlation between resilience and satisfaction could arise at this point. Most control variables are time constant, such as gender or highest parental education. The only time varying control variables are the field of study and whether the respondent lives with her family at the time of survey). While we are confident that most relevant confounding factors are accounted for, since only observational data are available, it is usually not possible to rule out all confounding. Consequently, we cannot argue that the results represent pure causal effects and regard them as associations instead. The reader can decide whether he or she believes how well these numbers represent causal effects or whether spurious influence might still be present that are not accounted for. All variables of the analyses are presented in Table 1 for a quick overview.

**Table 1:** Overview of all variables of the study

Treatment variable: resilience status in 2012			
Control variables (+ Wave dummies)			
Gender	Parental ISEI	Age in 2011	Number of siblings
Years in kindergarten	Age of father in 2011	Age of mother in 2011	University of Applied Sciences
Student loan eligibility	Mother has died	Father has died	Place of birth
Migration status	Field of study	Parental education level	Living with family
Dependent variables			
Life satisfaction	Intention to drop out	Average grades	

*Source* NEPS SC5

### Strategy of analysis

Since all outcome variables were measured at several points in time (panel design), longitudinal models can be applied. It should be noted that the central explanatory variable, the status of resilience, was measured in 2012 and is therefore constant over time. Technically, the following analyses are calculated as multilevel growth-curve models. This estimation procedure is mandatory, as otherwise standard errors would be incorrectly calculated based on correlating observations by the same person. Growth-curve models are chosen since the respondents were surveyed annually and within a time frame of a few weeks. Since there is little variation in the time of survey, other approaches like survival models cannot reveal further insight since these models require a finer-grained time of survey, for example, monthly or better even weekly with a large variation for all participants. Therefore growth-curve was selected, which give very similar results to panel regression models with random effects. Linear models are used for all dependent variables because the variables are scaled continuously (OLS regression technique). The models are built step by step: The first model contains only the explanatory variable resilience, the wave dummy, and their interactions to allow for most flexible estimation of effects for each wave. This form of parameterization is rather uncommon for growth-curve models, however, beneficial for the present data since only a small number of time points is available and all participants were surveyed at the same points in time (Rabe-Hesketh and Skrondal 2012). This design has some beneficial properties: due to the interaction effects, the change or growth of the outcomes is modeled independently for each point in time so that changes in all directions are mapped flexibly. Furthermore, in contrast to regular Growth-curve models, we do not have to make assumptions about the mathematical shape of the curve, for example, linear or exponential. The results are therefore almost free of model assumptions. Hence, this approach adds further flexibility and looses artificial constraints. The second model adds all control variables. For mathematical precision, the model equation for the models without control variables is shown below. The last two terms describe the person-specific (j) and observation-specific (ij) error terms. The variable resilience varies only between persons but not within a person, while the wave dummies vary only within a person but not between persons.

$$\begin{array}{c}{Y}_{ij}={\beta }_{0}+{\beta }_{1}{Resil}_{j}+{\beta }_{2}W{1}_{i}+{\beta }_{3}W{2}_{i}+{\beta }_{4}W{3}_{i}+{\beta }_{5}W{4}_{i}\\ +{\beta }_{6}{Resil}_{j}\times W{1}_{i}+{\beta }_{7}{Resil}_{j}\times W{2}_{i}+{\beta }_{8}{Resil}_{j}\times W{3}_{i}+{\beta }_{9}{Resil}_{j}\times W{4}_{i}+{u}_{j}+{\varepsilon }_{ij}\end{array}$$

We argue that, given the current data, this model design is preferable to inspect the temporal development of outcomes in interaction with the resilience status. Furthermore, graphs can be produced that allow an intuitive understanding of the developments, which is beneficial for interpretation and discussion. All analyses are carried out using Stata 16.1. Missing information was imputed using Multiple imputation by chained equations (MICE; 40 imputations after a burn-in sequence of 80) (Allison 2001; Azur et al. 2011). Typical diagnostic tests such as convergence of imputation models were examined. Graphs are generated using the software package *mimrgns* (Klein 2014).

## Results

### Descriptive statistics

A good description should be the foundation of all advanced analyses. Consequently, some basic descriptive statistics are presented in Table 2. All values are computed for wave two (measured in 2012).

**Table 2:** Descriptive statistics

	Mean	SD	Median	Min	Max
Resilience	0.15	0.36	0.00	0.00	1.00
Female	0.60	0.49	1.00	0.00	1.00
Parental ISEI	53.54	18.95	54.11	12.00	88.00
Age in 2011	21.94	2.47	21.00	17.00	35.00
Number of siblings	1.50	1.11	1.00	0.00	8.00
Years in kindergarten	2.96	0.96	3.00	0.00	13.00
Age of father in 2011	53.32	6.20	53.00	25.63	94.81
Age of mother in 2011	50.49	5.26	50.00	31.00	92.00
University of Applied Sciences	0.24	0.43	0.00	0.00	1.00
Student loan eligible	0.43	0.50	0.00	0.00	1.00
Mother has died	0.01	0.11	0.00	0.00	1.00
Father has died	0.03	0.18	0.00	0.00	1.00
Place of birth					
Germany, West	0.74	0.44	1.00	0.00	1.00
Germany, East	0.20	0.40	0.00	0.00	1.00
Abroad	0.06	0.24	0.00	0.00	1.00
Migration background					
Both parents born in Germany	0.84	0.37	1.00	0.00	1.00
One parent born abroad	0.06	0.25	0.00	0.00	1.00
Both parents born abroad	0.10	0.30	0.00	0.00	1.00
Field of study*					
Cultural sciences/humanities	0.28	0.45	0.00	0.00	1.00
Economics/law/social sciences	0.26	0.44	0.00	0.00	1.00
STEM	0.24	0.43	0.00	0.00	1.00
Health sciences/medicine	0.05	0.22	0.00	0.00	1.00
Engineering	0.15	0.36	0.00	0.00	1.00
Arts	0.03	0.16	0.00	0.00	1.00
Parental education					
Low	0.10	0.31	0.00	0.00	1.00
Medium	0.28	0.45	0.00	0.00	1.00
High	0.27	0.44	0.00	0.00	1.00
University	0.35	0.48	0.00	0.00	1.00
Living with family*	0.46	0.50	0.00	0.00	1.00
Intention to drop out*	1.56	0.56	1.40	1.00	4.00
Average grades*	2.72	0.55	2.73	1.00	4.00
Satisfaction*	7.16	1.65	7.50	0.00	10.00

*Source* NEPS SC5, imputed data. Statistics calculated for 2012

Variables marked with an asterisk are not time constant and can change in the subsequent waves of the survey

**Fig. 1:**
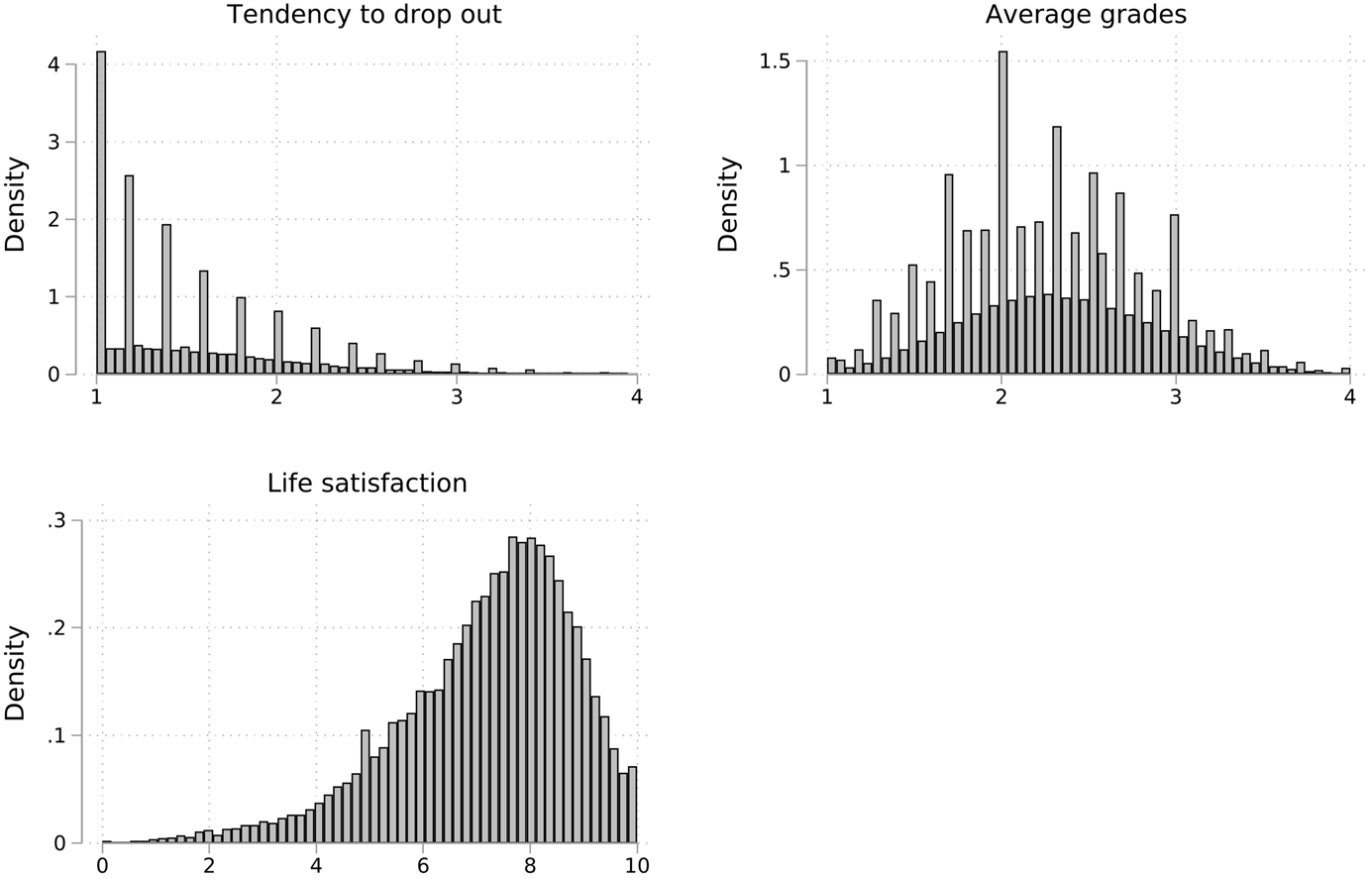
Distribution of outcome variables in 2012. *Source* NEPS SC5, imputed data. Higher numerical values represent a higher tendency to drop out, better grades and a higher life satisfaction

For binary variables, the mean can be interpreted as a share. We learn that about 15% of the sample is classified as resilient. About 60% of all participants are female and the age in 2011 was about 22 years. 24% start their studies at a university of applied sciences, 43% are eligible for student loans and about 84% of students do not have a migration background. Of interest are especially some characteristics of the families. We see that the sample is rather selective and more than 60% of the sample have highly educated parents. This displays that attending tertiary education and social origin are quite related in Germany, which is relevant when the findings are generalized to a wider population and possibly other countries. Additionally to the numerical statistics, the distribution of the central outcome variables is also depicted graphically using histograms in Fig. 1.

## Longitudinal analyses

**Fig. 2:**
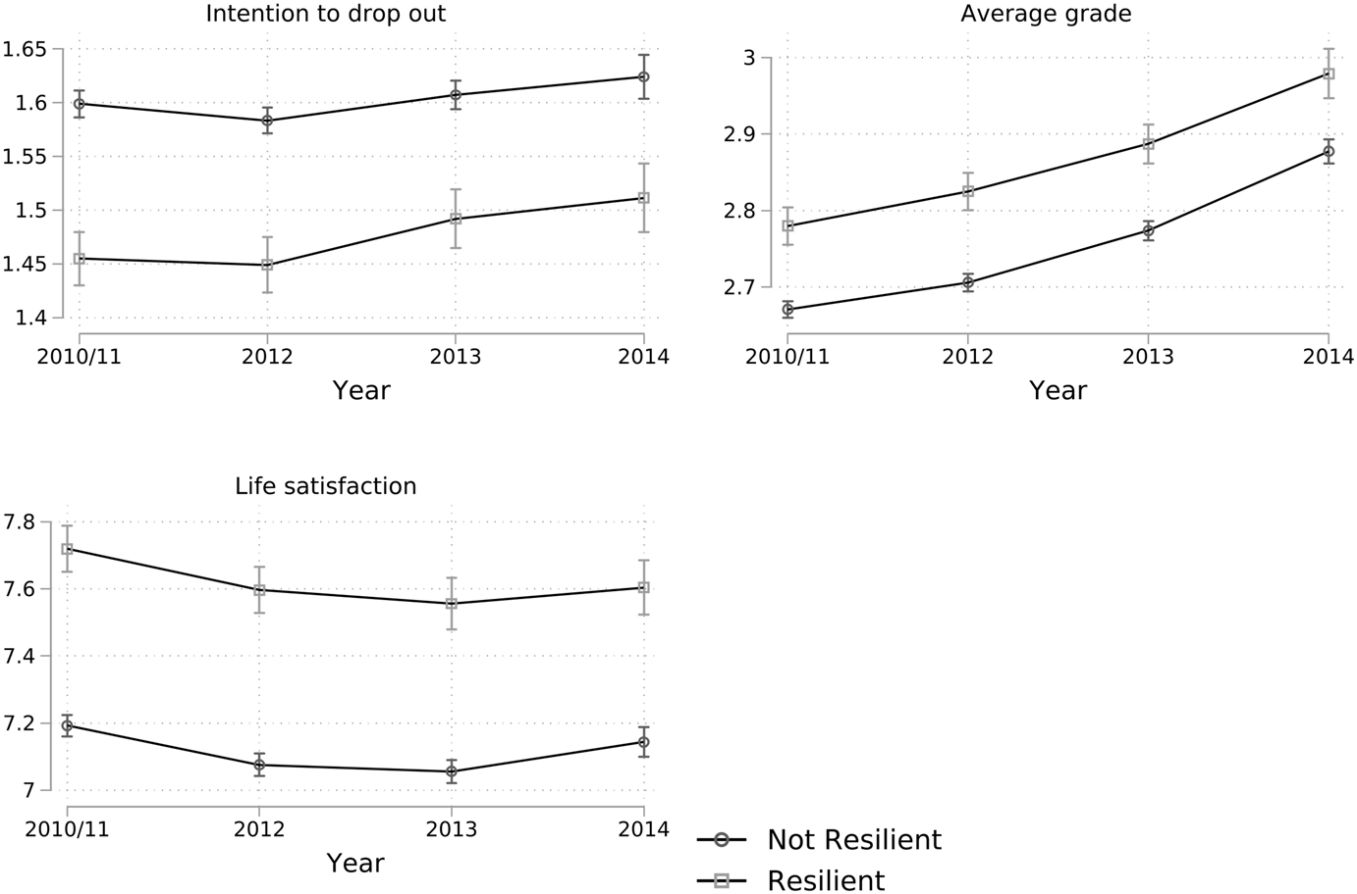
Temporal development of outcomes by resilience status. *Source* NEPS SC5, imputed data. Confidence bars are based on 95% confidence intervals

The regression results are reported in Table 3. It is clear that the resilience dummy has a highly significant effect in all models. Due to the numerous interactions, an interpretation of the pure numbers is not very clear, so that predicted outcomes are calculated for a better understanding in Fig. 2. These then refer to the complete models with all interactions and control variables and are broken down by wave and resilience status. The effects of resilience are positive in all cases (lower intention to drop out, higher grades and higher life satisfaction).

**Table 3:** Results of longitudinal growth-curve models

	Intention to drop out		Average grades		Life satisfaction	
	M1	M2	M3	M4	M5	M6
Resilience	− 0.144***	− 0.139***	0.109***	0.090***	0.526***	0.511***
	(0.014)	(0.014)	(0.014)	(0.014)	(0.040)	(0.040)
Female		− 0.021*		0.092***		− 0.035
		(0.009)		(0.010)		(0.024)
Age in 2011		0.008***		− 0.016***		− 0.062***
		(0.002)		(0.002)		(0.006)
Place of birth						
Western Germany		Ref		Ref		Ref
Eastern Germany		− 0.012		− 0.035**		− 0.054
		(0.011)		(0.013)		(0.030)
Foreign-born		0.032		− 0.011		− 0.091
		(0.023)		(0.026)		(0.064)
Migration status of parents						
Both parents born in Germany		Ref		Ref		Ref
One parent born abroad		0.070***		− 0.068***		− 0.093*
		(0.019)		(0.019)		(0.045)
Both parents born abroad		0.052**		− 0.160***		− 0.078
		(0.020)		(0.023)		(0.053)
University of Applied Sciences		− 0.036**		0.132***		0.026
		(0.011)		(0.012)		(0.028)
Field of study						
Cultural sciences		Ref		Ref		Ref
Law/economics/social sciences		0.060***		− 0.141***		− 0.120***
		(0.012)		(0.014)		(0.031)
STEM		0.035**		− 0.170***		− 0.041
		(0.011)		(0.013)		(0.030)
Medicine/health		− 0.069***		− 0.137***		0.138**
		(0.018)		(0.024)		(0.048)
Engineering		0.033*		− 0.303***		− 0.067
		(0.015)		(0.015)		(0.038)
Arts		0.008		0.123***		0.036
		(0.025)		(0.028)		(0.070)
Average parental ISEI		− 0.000		0.001***		0.004***
		(0.000)		(0.000)		(0.001)
Student loan eligible		0.022*		− 0.038***		− 0.239***
		(0.010)		(0.009)		(0.024)
Parental education level						
Low		Ref		Ref		Ref
Medium		− 0.006		0.024		0.053
		(0.015)		(0.017)		(0.045)
High		− 0.011		0.039*		0.040
		(0.016)		(0.017)		(0.044)
Tertiary		− 0.009		0.049*		0.059
		(0.017)		(0.021)		(0.049)
Number of siblings		0.011**		− 0.008		0.010
		(0.004)		(0.004)		(0.010)
Years in kindergarten		− 0.004		− 0.009*		− 0.008
		(0.005)		(0.004)		(0.012)
Mother has died		− 0.009		0.000		− 0.079
		(0.042)		(0.039)		(0.099)
Father has died		− 0.004		0.023		− 0.121*
		(0.024)		(0.027)		(0.061)
Age of father in 2011		0.002		− 0.001		− 0.005
		(0.001)		(0.001)		(0.003)
Age of mother in 2011		− 0.000		0.002		− 0.003
		(0.001)		(0.001)		(0.003)
Living with family		0.053***		− 0.025*		− 0.098***
		(0.009)		(0.011)		(0.020)
Constant	1.603***	1.315***	2.668***	2.961***	7.185***	8.947***
	(0.006)	(0.063)	(0.006)	(0.068)	(0.016)	(0.174)
Observations	70,040	70,040	70,040	70,040	70,040	70,040
Persons	17,510	17,510	17,510	17,510	17,510	17,510
Adjusted *R*^2^	0.007	0.021	0.025	0.116	0.014	0.041

*Source* NEPS SC5, imputed data. Standard errors in parentheses. Adjusted *R*^2^ statistics are taken from OLS regression models and are to be seen as approximations. All models include wave dummies and wave resilience interaction variables

*p < 0.05; **p < 0.01; ***p < 0.001

The computations clearly demonstrate the positive effects of resilience in Table 3. Since the effect of resilience is quite constant over time, Table 3 reports the *overall* effect (that is the effect summarized for the entire observational window). For example, when we look at Model M1, we see that the coefficient of resilience is − 0.144. This means that students with a high resilience have an intention to drop out that is about 0.144 points lower than for students with a low resilience. Adding all control variables in model M2 basically does not change the coefficient. This finding suggests that resilience and intention to drop out are negatively associated and students with high resilience have, on average, a lower intention to drop out of their studies. The results in the other models are very similar. Especially the effect for life satisfaction is impressive as it is above 0.51 points (M6), which is quite large on a scale from 0 to 10. The graphical representation of the results visualizes the developments over time and is a bit more flexible since not the overall effect is summarized but for every year individually. However, results are rather stable with the only exception grades, which tend to become better over the course of studies which is expected due to various reasons (e.g., dropout of the worst performers, just to name one). With respect to the resilience variable, the positive effects are highly stable and the difference between the two groups does hardly ever change over the years.

## Discussion and conclusion

The primary objective of the paper is to investigate the relationship between resilience and academic success. The analyses show a clear effect: Students with resilience status consistently display significantly more positive results over the entire time and with regard to all three outcomes. Statistically speaking, these results are very strong since the confidence bands never overlap. The numerical results indeed reveal that all effects are always highly significant with small p-values. Since a large number of relevant control variables is included and thus the occurrence of spurious correlations is unlikely, hypothesis 1 is accepted, which means that resilient students do have better outcomes, on average. The results of the study are in line with theoretical expectations and previous research findings (Allan et al. 2014; Esquivel et al. 2011; Hartley 2011) The present contribution therefore demonstrates the positive and protective effects of resilience for persons studying in Germany. The effect of resilience is approximately constant over the entire time span of four years, which underlines the temporal stability of the protective effect. Methodologically, the analyses go beyond most comparable studies by establishing a clear temporal order using longitudinal data and by using context data to rule out inverse causality. It therefore contributes to the state of research on resilience and study trajectories and proves that resilience is a positive characteristic or disposition. While results of the statistical models are quite clear, they deserve some more discussion. Firstly, as already mentioned before, we cannot interpret these findings as pure causal effects since only observational data are at hand and even under the inclusion of multiple relevant controls, one can never statistically “prove” causality and rule out further “hidden” confounding pathways. Consequently, we conservatively consider these results as associational instead, which is nevertheless relevant. Even if these are only associations, they can still be beneficial for students and universities alike. For example, since apparently students with low values of resilience display worse outcomes on average, screening and finding these students would be an option to support them individually. As this is a high-risk group for dropout, just to mention one outcome, if one could identify these students early in the course of their studies, additional support could reduce their dropout probabilities, which would be in the interest of the educational institutions. Consequently, even if only associations are revealed, they can still be important for intervention. If one goes beyond this and assumes that (some) causal effects are present, offering resilience-training could even be an option to increase resilience in the student population and get overall positive effects from this. Indeed, some universities already implemented some programs and offer opportunities to strengthen resilience in a targeted manner (“Florida State University – Student Resilience Project” n.d.; Marthers 2017) with positive outcomes (Sood et al. 2011; Zamirinejad et al. 2014). If it were possible to train resilience in this way, this could be a sensible approach to increase the success rate of university students. Whether and to what extent this is possible must be clarified by subsequent investigations. Especially the emergence of COVID-19, which impacted the world like no other crisis in the last 50 years can be regarded as a major external shock that is related to resilience. The basic assumption is that resilient students are better able to deal with the negative experiences of the crisis and develop strategies to still maintain a high level of performance in their studies. While these aspects cannot be studied with the data at hand, this appears to be a highly interesting option for further research. How does COVID-19 affect students and performance in tertiary education and are resilient students able to mitigate these negative effects? Hopefully, new data sources can open up this area of research that seems very promising for resilience research. This is also an interesting option to investigate issues of causality, since COVID-19 is a random and external “shock” and does not have confounding factors.

Finally, the limitations of the own investigations will be discussed. Challenges such as panel attrition, which makes inference fundamentally more difficult, should be mentioned here. The permanent survey dropout reduces the power of all analyses and can generate bias, which is difficult to control. A solution using statistical weights is not yet possible, as these are simply not yet developed for selective dropout, which is due to the complexity of the data. Nor does the NEPS Methodological Council currently make any general recommendations regarding sampling weights (Zinn et al. 2017, p. 12). However, it is generally the case that analyses that are more causally than descriptively oriented tend to be less affected by this problem. Since values are also imputed, we hope that the problem of missing values is ameliorated. Furthermore, it is open to criticism that the operationalization of resilience is only one of many possibilities and is due to the available data basis. Comparisons with other, ideally continuously scaled measures would be desirable here. Another question is about the external validity. We argue that most results found with the German dataset will probably also be valid for other countries since tertiary education has been adapted through the Bologna Process in the last decades and in 2020, basically all studies have switched to the BA/MA system. Since this means that the tertiary system is nowadays quite similar to other countries and that resilience is a psychological trait that is rather stable, we believe that our findings will probably hold for most other comparable countries, at least highly developed ones in western, industrialized nations. However, as mentioned in brief above, students in the sample often have highly educated parents, which might be different in other countries (relation of tertiary education and social origin). To test this, we invite replication and addition studies, optimally in a multinational setting. Lastly it should also be kept in mind that the population of the analyses consisted of people of age below 35 at the start of their studies. While this is the typical population of the German tertiary system, it is unclear whether the same conclusions hold for older participants. They have a different position in the society than absolute beginners and probably also different psychological traits and characters. Therefore, all conclusions only hold for the younger group in German tertiary education.

## Data Availability

Data are available from the German Leibniz Institute for Educational Trajectories (www.lifbi.de). Code is available from the authors upon request.

## Footnotes

1 The following quotations are examples of such a consideration: „Resilient individuals tend to show high motivation in coping with negative life events… “ (Terzi 2013, p. 103); „(…) concluded that resilient people were positive about life (…)“ (Wang 2009, p. 26); „(…) to ascertain the differences between resilient and nonresilient individual[s]“ (Zuill 2016, p. 15f.). In all cases it is clear that resilience is understood as a (stable) disposition.

2 This paper uses data from the National Educational Panel Study (NEPS): Starting Cohort First-Year Students, https://doi.org/10.5157/NEPS:SC5:12.0.0. From 2008 to 2013, NEPS data were collected as part of the Framework Program for the Promotion of Empirical Educational Research funded by the German Federal Ministry of Education and Research (BMBF). As of 2014, NEPS is carried out by the Leibniz Institute for Educational Trajectories (LIfBi) at the University of Bamberg in cooperation with a nationwide network.
